# Ecological momentary assessment to uncover affect dynamics in patients with hereditary angioedema: a complementary tool for enhancing quality of life evaluation

**DOI:** 10.3389/fpsyg.2026.1799040

**Published:** 2026-08-07

**Authors:** Luca Ranucci, Alessandra Gorini, Monica Parati, Azzurra Cesoni Marcelli, Lorenza Chiara Zingale, Beatrice De Maria, Clara Gino, Aida Zulueta, Riccardo Sideri, Riccardo Senter, Francesca Perego

**Affiliations:** 1 Istituti Clinici Scientifici Maugeri IRCCS, Milan, Italy; 2 Dipartimento di Scienze Cliniche e di Comunità, Dipartimento di Eccellenza 2023-2017, Università degli Studi di Milano, Milan, Italy; 3 Dipartimento di Medicina dei Sistemi, Ospedale Universitario di Padova, Padua, Italy

**Keywords:** affect, burden of disease, ecological momentary assessment, hereditary angioedema, quality of life, rare disease

## Abstract

**Introduction:**

Hereditary Angioedema due to C1-INH deficiency (HAE-C1INH) is a rare genetic condition associated with poor quality of life (QoL). Affect, defined by Activation (energy) and Valence (pleasantness), is linked to QoL and fluctuates daily, reflecting emotion regulation processes. This study explored affective dynamics in HAE-C1INH patients, with (HAE A+) and without attacks (HAE A−), comparing them to healthy controls (CTR), using Ecological Momentary Assessment (EMA). Secondarily, correlation between affective dynamics and Angioedema Quality of Life (AE-QoL) was assessed.

**Methods:**

In this observational study, 12 HAE-C1INH patients and 14 CTR completed weekly EMA surveys over 16 weeks, assessing Positive Activation (PA), Negative Activation (NA), and Valence (VA). Affective intensity, variability, and instability were computed. HAE-C1INH participants also completed two self-administered questionnaires: AE-QoL and Angioedema Control Test (AECT) assessing QoL and disease control, respectively.

**Results:**

Patients showed higher mean VA (*p* = 0.016) and lower NA for worry (*p* = 0.021) than CTR. HAE A− had higher PA and VA, and lower NA for anger and worry than both HAE A+ and CTR. AE-QoL correlated negatively with PA (r = −0.78, *p* = 0.003) and VA (r = −0.68, *p* = 0.014).

**Conclusion:**

Patients, especially those without attacks, displayed more positive emotional profiles than controls. EMA captured emotional patterns not evident in traditional questionnaires, supporting its value as a complementary tool for QoL evaluation in rare chronic diseases.

## Introduction

Hereditary angioedema due to C1-INH deficiency (HAE-C1INH) is a rare genetic disorder characterized by increased vascular permeability and consequent unpredictable swelling of subcutaneous and/or submucosal tissues (Wu et al., 2016; De Maat et al., 2018; Zuraw et al., 2025). The most common causes are mutations in the SERPING1 gene (Reshef et al., 2024), resulting in either a deficiency (HAE-C1INH type 1) or a dysfunction (HAE-C1INH type 2) of the C1 inhibitor protein. These unpredictable swellings can affect the extremities, face, and gastrointestinal tract, and can be life-threatening during upper airway attacks (Rajan et al., 2021), significantly affecting functional capacity and the overall quality of life (QoL) (Chong-Neto, 2023).

The unpredictable nature of these attacks and their recurrent nature, combined with the need for ongoing disease management and the psychosocial impact of a rare disease, can significantly compromise patients’ resilience and adaptive capacity (Lumry and Settipane, 2020), ultimately contributing to a state of increased vulnerability (Caballero et al., 2014) arising from the depletion of physiological and psychological reserves and imposing a multifaceted physical and psychological burden (Lumry et al., 2010).

In recent years, the therapeutic landscape of HAE-C1INH has evolved substantially: alongside on-demand therapy, modern long-term prophylaxis, including plasma kallikrein inhibitors such as lanadelumab and berotralstat, has markedly improved disease control, reducing the frequency and severity of attacks and improving QoL. Nonetheless, how effective disease control translates into patients’ day-to-day affective experience remains largely unexplored.

To date, the burden of HAE-C1INH has been exclusively assessed using specific QoL questionnaires, like the Hereditary Angioedema Quality of Life (HAE-QoL) (Prior et al., 2012) or the Angioedema Quality of Life (AE-QoL) (Weller et al., 2012), which evaluate aspects such as physical and mental health, social relationships, and daily functioning. While these instruments provide valuable insights into the overall impact of the disease and are instrumental in measuring *treatment* effectiveness (Prior et al., 2012), they present significant limitations. In particular, they fail to capture fluctuations over time and are susceptible to recall bias, which risks to compromise their ecological validity (Jager et al., 2020). Moreover, QoL is closely and bidirectionally related to changes in affect which cannot be measured through traditional psychological questionnaires (Lima et al., 2023; Pirla et al., 2023; Houben et al., 2015). Affect encompasses the full range of emotional experiences (Watson and Tellegen, 1985; Cacioppo and Berntson, 1999) and, according to Russell’s Circumplex Model (Russell, 1980), can be defined along two dimensions: Arousal (or Activation), and Valence. Arousal refers to the energy level associated with an emotion, ranging from low (e.g., calmness) to high (e.g., excitement), while Valence indicates the perceived pleasantness (e.g., happiness) or unpleasantness (e.g., sadness) of the emotional experience (Russell, 1980). Affect shows remarkable short-term variability (Röcke and Brose, 2013), whose dynamics and stability, indicating the individual emotion-regulatory processes, can be assessed only using repeated evaluations in real-life contexts, instead of traditional psychological questionnaires.

Ecological Momentary Assessments (EMAs) represent an innovative methodological approach (Shiffman et al., 2008) to collect real-time data in the individuals’ natural environments, enabling continuous monitoring over time. This approach not only minimizes recall bias, but also enhances ecological validity, providing a more nuanced understanding of the responders’ daily experiences (Shiffman et al., 2008).

To date, EMAs have been also successfully implemented in a variety of clinical contexts (Tutunji et al., 2023; Vansimaeys et al., 2017; van Os et al., 2014). In HAE-C1INH specifically, our research group showed, in a dedicated feasibility and acceptability study on the same sample, that a 16-week weekly EMA protocol is highly feasible and well accepted by patients, achieving results similar to healthy individuals (Parati et al., 2026).

Building upon these considerations, the main aim of this study is to explore the affective dynamics in a sample of HAE-C1INH patients and to compare them with those occurring in a sample of healthy controls (CTR). The secondary aim is to analyse the influence of attacks on the affective variations by comparing patient’s with attacks (HAE A+), defined as those who reported at least one attack during the study period, with those without (HAE A−) and to correlate the affect composite scores with the AE-QoL in the patient sample.

## Methods

The study was approved (approval number: 2651 CE, 29 June 2022) by the Ethics Committee of Istituti Clinici Scientifici Maugeri (Pavia, Italy). All research was performed in accordance with relevant guidelines and regulations, it was conducted in accordance with the Declaration of Helsinki, and all participants provided written informed consent for all the study procedures prior to the enrollment visit.

### Study design and population

This observational prospective study enrolled a sample of 12 HAE-C1INH patients, consecutively enrolled during routine clinical visits, from the Italian Network of Hereditary and Acquired Angioedema (ITACA) who referred for clinical treatments to a tertiary care center (IRCCS Istituti Clinici Scientifici Maugeri) in Milan (Italy) and 14 healthy individuals, recruited among hospital staff and their personal network.

Given the rarity of the disease, and the lack of prior data on affect dynamics in this population, a formal *a priori* sample size calculation was not feasible, as no reliable effect-size estimate was available. The repeated longitudinal design was nonetheless expected to provide more stable and reliable individual-level affect estimates.

The study was conducted between September 2023 and January 2024, and shares the same sample and protocol with a previous study assessing the feasibility and acceptability of a longitudinal digital monitoring in HAE-C1INH (Parati et al., 2026). Inclusion criteria for both patients and CTR were to be older than 18 years, speaking and understanding Italian, and to have at least one electronic device (e.g., smartphone, tablet, computer). Individuals with cognitive impairment, mental illness and the inability to sign the informed consent were excluded from the study.

Patients underwent a clinical assessment by clinician to collect information about the type of diagnosis, the use of long-term prophylactic treatments (LTP), and the occurrence of attacks during the observation period. Participants with major comorbidities known to significantly affect QoL, such as active malignancies, severe cardiovascular or chronic kidney disease, or other disabling chronic conditions were not enrolled, so as to limit their potential confounding effect.

This report conformed to the Strengthening of the Reporting of Observational Studies (www.strobe-statement.org).

### Data collection and measurement

Once enrolled, patients completed the Angioedema Control Test (AECT) (Weller et al., 2020) and the Angioedema Quality of Life (AE-QoL) questionnaires (Weller et al., 2012). The AECT is a 4-item Patient-Reported Outcome (PRO) questionnaire designed to assess angioedema control in patients with recurrent angioedema, including HAE-C1INH. The AECT score ranges from 0 to 16, with higher scores indicating effective disease control. AE-QoL consists of 17-item PRO questionnaire with a four-domain structure (functioning, fatigue/mood, fears/shame, food) developed to measure health-related QoL impairment in patients with recurrent angioedema. Higher scores indicate greater QoL impairment.

Participants where then registered in the REDCap™ system (Harris et al., 2009), which would automatically send weekly and monthly prompts (detailed below) for 16 consecutive weeks.

### Monthly psychological assessments

All participants were asked to complete the Generalized Anxiety Disorder-2 (GAD-2) and the Patient Health Questionnaire-2 (PHQ-2) once a month, for a total amount of 4 times (Harris et al., 2009). These questionnaires assess how often an individual has experienced two core symptoms of anxiety and depression over the past 2 weeks, respectively (Spitzer et al., 2006; Spitzer, 1999). Both scales have a score range from 0 to 6. A PHQ-2 score ≥2 and a GAD-2 score ≥3 indicate clinically relevant symptoms of depression and anxiety (Giuliani et al., 2021).

### Weekly EMA

Emotional states were assessed weekly for all participants over 16 consecutive weeks using the EMA approach. Each evaluation was composed of 10 items taken from the “Positive Activation, Negative Activation and Valence – Short Form scale (PANAVA-KS)” (Schallberger, 2000), a validated tool to assess affect by measuring positive activation (PA), negative activation (NA) and Valence (VA) related to different moods. To make completion more intuitive, validated emoticons specifically developed for non-verbal evaluations in EMA studies (Schreiber and Jenny, 2020) were placed next to the name of the different moods.

**Figure 1:**
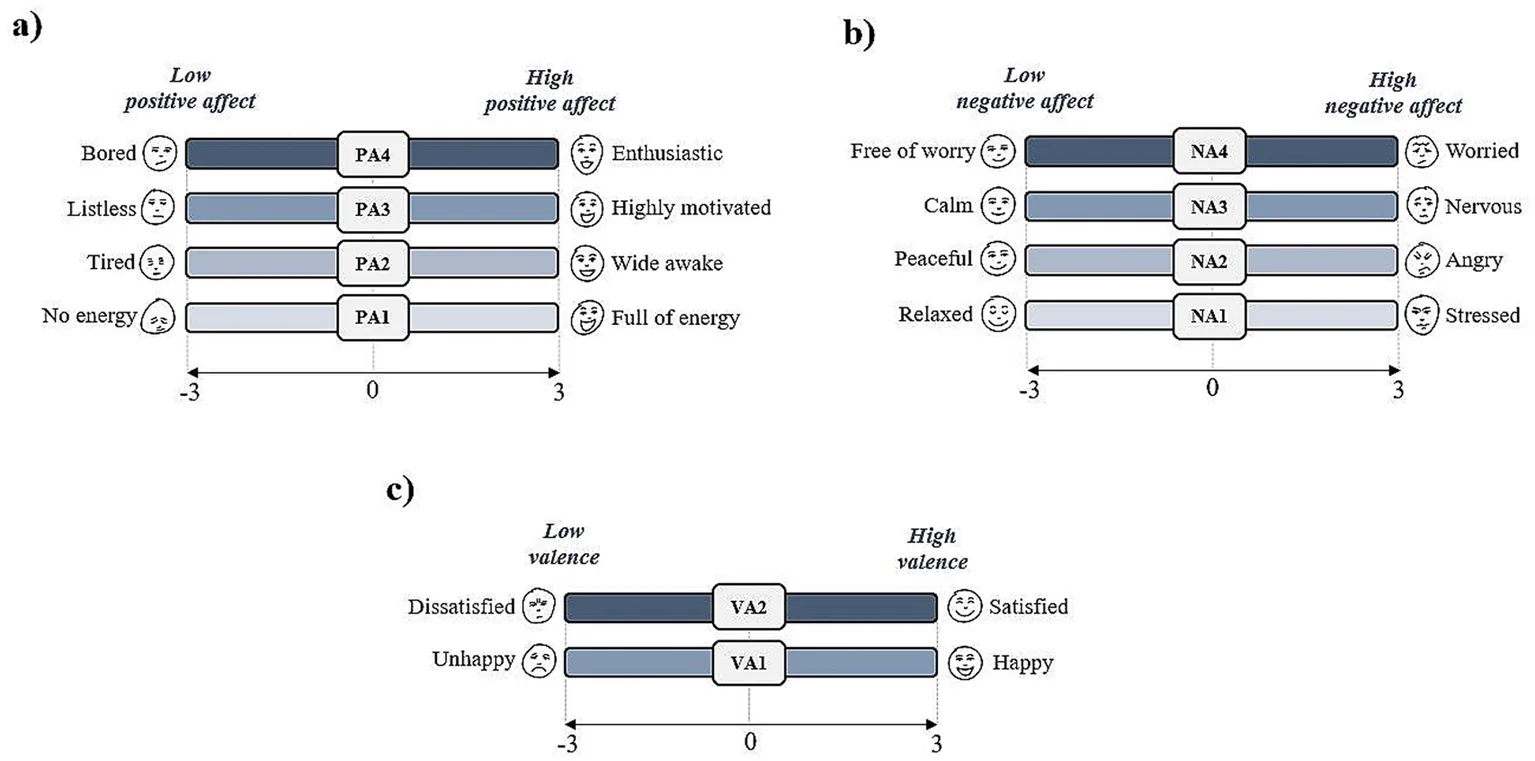
Schematic representation of the examined items and the scoring modalities related to positive affect **(a)**, negative affect **(b)**, and valence **(c)**.

For each item, participants were instructed to indicate their affect state at the moment of the evaluation by moving a slider’s cursor closer to their perceived state. Item endpoint scores ranged from −3 to 3, with the highest score indicating the highest level of affect experienced. The list of items is presented in the graphical example of the items is presented in Figure 1. EMA prompts were sent to the participants’ electronic device according to a specific scheduling and responders were asked to answer within 90 min of receipt.

The study design and procedures have been previously described in detail elsewhere (Parati et al., 2026).

### Measures of emotion dynamics

To evaluate affective dynamics, three key measures were computed (Jahng et al., 2008; Schneider et al., 2020): affective intensity, affective variability and affective instability. These measures capture three complementary and non-redundant dimensions of affective experience: the average level of affect (intensity), the magnitude of fluctuations around that level (variability), and the magnitude changes between successive assessments (instability). These calculations were performed separately for each EMA item.

#### Affective intensity

Individual’s affective intensity (*μ*) represents the average of the respondents’ rating to each EMA item across the different time points. The mean (μ) computed across the four PA items was labelled as μ_PA1_, μ_PA2_, μ_PA3_, and μ_PA4_, while the mean computed across the four NA items was labelled as μ_NA1_, μ_NA2_, μ_NA3_ and μ_NA4_. For VA, the mean was calculated over two items and referred as μ_VA1_ and μ_VA2_. It was calculated as follows:


$$\mu =\frac{1}{N}\sum_{i=1}^{N}x_{i}$$

where $x_{i}$ is the *i*^th^ rating of a given subject with *i* ranging from 1 to *N*. *N* was equal to 16 weeks.

#### Affective variability

Individual’s temporal variability (ϭ^2^) represents the within-person variance of $x_{i}\;$ across the observation period. Temporal variability captures the magnitude of fluctuations in a respondent’s affective states around the average affect level and was calculated as follow:


$$\sigma^{2}=\frac{1}{N-1}\sum_{i=1}^{N}{\left(x_{i}-M\right)}^{2}$$

where $x_{i}$ is the *i*^th^ rating of a given subject with *i* ranging from 1 to N, and M is the mean affect reported by each individual for the considered item. Variability indices follow the same nomenclature used for affective intensity.

#### Affective instability

Temporal affective instability (MSSD) was calculated by taking the Mean of the Squared Successive Differences between ratings $(x_{i+1}-x_{i})\;$ for each respondent across the observation period. Affective instability refers to the magnitude of shifts (both increases and decreases) in affect levels and was calculated as follows:


$$\text{MSSD}=\frac{1}{N-1}\sum_{i=1}^{N-1}{\left(x_{i+1}-x_{i}\right)}^{2}$$

MSSD labels followed the same nomenclature as previously described.

### Composite indices

For each subject, composite indices of *μ*, σ^2^ and MSSD were obtained by computing the mean of each key measure across the corresponding items (4 for PA, 4 for NA and 2 for VA). The composite measures of PA, NA and VA related to μ were named μ_PA_, μ_NA_ and μ_VA_ respectively. Similarly, σ^2^_PA_, σ^2^_NA_, σ^2^_VA_, and MSSD_PA_, MSSD_NA_, and MSSD_VA_ were obtained.

### Data analysis

Descriptive statistics were used to describe sociodemographic, clinical and psychological characteristics, and EMA features in the HAE-C1INH and CTR groups and subgroups (i.e., HAE A+ and HAE A−). Continuous variables were reported as median [interquartile range], and dichotomous variables as absolute values. The Wilcoxon rank-sum test or Fisher’s Exact Test was applied to continuous or dichotomous variables to test for possible significant differences between HAE and CTR groups, and to compare HAE A+ with HAE A− group, HAE A+ with CTR group, and HAE A− with CTR group. Correlation analyses using Spearman’s Rho correlation coefficient ( ρ) were performed on continuous variables to measure the strength of the associations between EMA measures and clinical scales. Correlations were interpreted as very strong when | ρ | ≥ 0.70, strong when 0.40 ≤ | ρ | ≤ 0.69, moderate when 0.30 ≤ | ρ | ≤ 0.39, weak when 0.20 ≤ | ρ | ≤ 0.29, negligible when 0 ≤ | ρ | ≤ 0.19 (Dancey and Reidy, 2007).

Given the exploratory nature of this study, no post-hoc correction for multiple comparisons was made, therefore, *p*-values should be interpreted as exploratory.

## Results

### Descriptive characteristics of the study samples

Twelve HAE-C1INH (females *n* = 7, age: 49.5 [21.8] years) and 14 CTR (females *n* = 8, age 30.0 [33.0] years) received EMAs. Demographic and clinical characteristics of the participants are reported in Table 1. Although the median age differed between groups, this difference was not statistically significant (*p* = 0.268).

**Table 1:** Demographic and clinical characteristics of the studied population.

Variables	HAE A+	HAE A−	HAE-C1INH	CTR
	(*n* = 7)	(*n* = 5)	(*n* = 12)	(*n* = 14)
Age (Years)	50.0 [27.5]	49.0 [15.0]	49.5 [21.8]	30.0 [33.0]
Sex (Male/Female)	2/5	3/2	5/7	6/8
Education (Years)	13.0 [4.0]	18.0 [5.0]	14.5 [5.0]	18.0 [3.3]
Diagnosis (Type I/II)	6/1	4/1	10/2	n.a.
Attacks (Yes/No)	7/0	0/5	7/5	n.a.
LTP (Yes/No)	3/4	5/0	8/4	n.a.
PHQ-2 (score)	1.5 [0.5]	0.3 [0.8]^b^	1.1 [1.3]	2.0 [1.6]
GAD-2 (score)	1.8 [1.3]	1.0 [1.3]	1.6 [1.4]	2.3 [1.6]
AECT (score)	14.0 [2.0]^a^	16.0 [0]	15.0 [2.3]	n.a.
AE-QoL (score)	34.0 [6.5]^a^	21.0 [4.0]	28 [13.5]	n.a.
AE-QoL Functioning (score)	4.0 [2.5]	1.0 [0]	4.0 [0.5]	n.a.
AE-QoL Fatigue/Mood (score)	11.0 [5.0]^a^	4.0 [0]	8.0 [6.3]	n.a.
AE-QoL Fears/Shame (score)	14.0 [7.5]	5.0 [2.0]	11.5 [7.5]	n.a.
AE-QoL Food (score)	2.0 [1.0]	10.0 [5.0]	2.0 [0.3]	n.a.

Data are reported as median [IQR]. HAE-C1INH, Patients affected by hereditary angioedema due to C1INH deficiency; HAE A+, patients with attacks; HAE A−, patients without attacks; CTR, Healthy controls; LTP, long-term prophylaxis; PHQ-2, Patient Health Questionnaire-2; GAD-2, Generalized Anxiety Disorder-2; AECT, Angioedema Control Test questionnaire; AE-QoL, Angioedema Quality of Life questionnaire. ^a^*p* < 0.05 HAE A+ vs. HAE A−; ^b^*p* < 0.05 HAE A− vs. CTR.

Ten patients had a type I diagnosis, two had a type II diagnosis, and most of them (*n* = 8) received LTP at the time of the evaluation, specifically lanadelumab with a posology of 300 mg every 4 weeks, for at least 1 year prior to enrolment. Seven patients had one or more attack during the study period. The HAE A+ group showed significantly lower scores in the AECT questionnaire (*p* = 0.042), and higher scores in the AE-QoL questionnaire (*p* = 0.034) and in its fatigue/mood subscale (*p* = 0.025) compared to the HAE A− group.

Both groups had GAD-2 scores below the clinical cut-off values indicating the absence of anxious symptoms. However, CTR obtained a PHQ-2 score at the lower limit of the clinical cut-off for mild depressive symptoms. Overall, there were no significant differences in GAD-2 and PHQ-2 scores except for PHQ-2 between HAE A− and CTR (*p* = 0.041), where the latter obtained higher scores.

### HAE-C1INH vs. CTR

**Figure 2:**
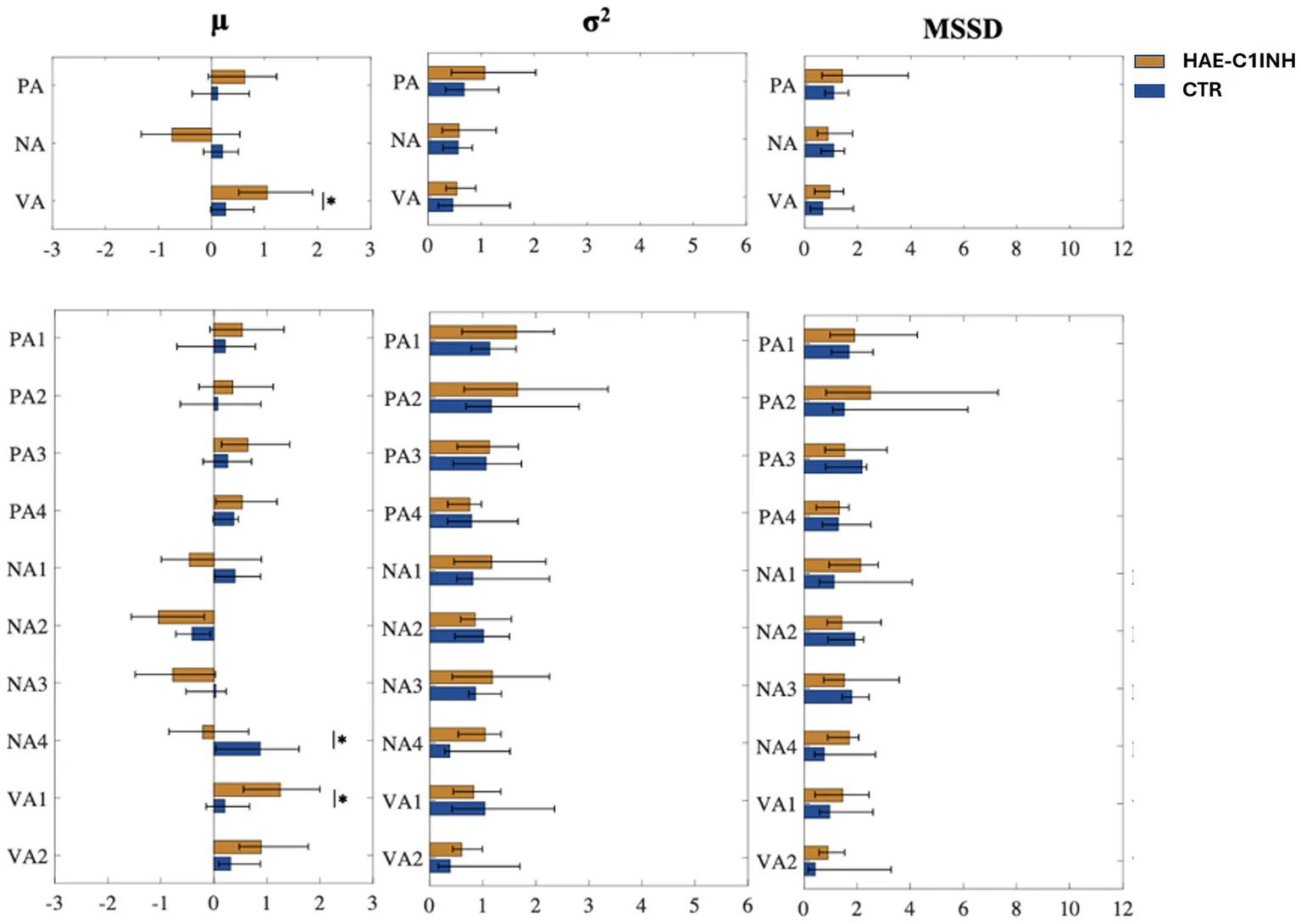
Comparison of the single items of the EMA features between HAE-C1INH and CTR group. *p < 0.05 HAE-C1INH vs. CTR.

Table 2 shows the EMA composite scores calculated in the HAE-C1INH and CTR groups. Figure 2 also shows the comparison between the groups in terms of the emotion dynamics derived from the individual items.

**Table 2:** Comparison of the scores between EMA features derived from patients with HAE-C1INH and those from CTR.

EMA	Measures	HAE-C1INH	CTR	*p*
		(*n* = 12)	(*n* = 14)	
Positive affect	μ_PA_	0.6 [1.3]	0.1 [1.0]	0.165
	σ^2^_PA_	1.1 [1.5]	0.7 [0.9]	0.572
	MSSD_PA_	1.4 [3.1]	1.1 [0.8]	0.643
Negative affect	μ_NA_	−0.7 [1.8]	0.2 [0.6]	0.181
	σ^2^_NA_	0.6 [0.8]	0.6 [0.5]	0.877
	MSSD_NA_	0.9 [1.1]	0.8 [2.2]	0.877
Valence	μ_VA_	1.1 [1.3]	0.3 [0.7]	0.016^a^
	σ^2^_VA_	0.6 [0.5]	0.5 [1.2]	0.959
	MSSD_VA_	1.0 [0.9]	0.7 [1.4]	0.797

Data are reported as Median [IQR]. HAE-C1INH, Patients affected by hereditary angioedema due to C1INH deficiency; CTR, Healthy controls; μ, Mean; σ2, Variance; MSSD, Mean of squared successive differences; PA, Positive activation; NA, Negative activation. VA, Valence. ^a^*p* < 0.05 HAE-C1INH vs. CTR.

There were no group differences in the positive and negative affect composite measures (*μ*, σ2, MSSD) (Table 2). However, the μ_NA4_ (free of worry/worried) was lower (less worried) in HAE-C1INH patients than in CTR (HAE-C1INH –0.2 [1.4] vs. CTR 0.9 [1.4], *p* = 0.021) (Figure 2).

A significant between-group difference was found in the composite mean Valence, which represents the pleasantness of an emotional experience (μ_VA_), with HAE-C1INH patients reporting higher values (HAE-C1INH 1.1 [1.3] vs. CTR 0.3 [0.7], *p* = 0.016) (Table 2). The main contribution to this finding is derived from the μ_VA1_ item (unhappy/happy) (HAE-C1INH 1.3 [1.3] vs. CTR 0.2 [0.8], *p* = 0.005), while the μ_VA2_ item (dissatisfied/satisfied) showed higher values in the patients group without reaching the statistically significant threshold (HAE-C1INH 0.9 [1.2] vs. CTR 0.3 [0.7], *p* = 0.051) (Figure 2). No differences were observed in σ^2^ and MSSD in the μ_VA_ (*p* > 0.542) and its individual items (*p* > 0.382) (Figure 2).

### HAE A+ vs. HAE A− vs. CTR

**Figure 3:**
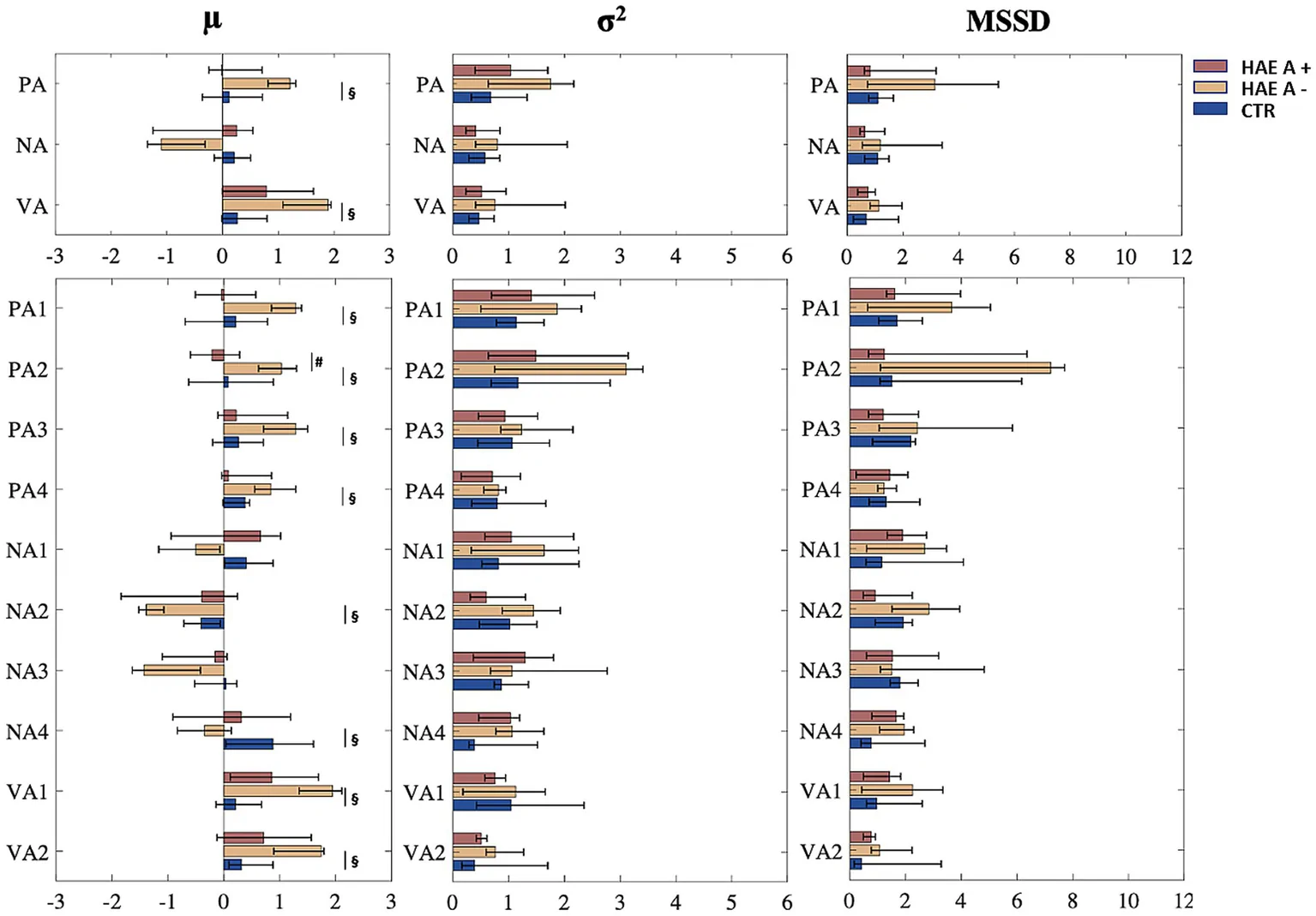
Subgroup analysis over EMA measures based on the occurrence of HAE-C1INH attacks. #*p* < 0.05 between HAE-C1INH patients with attacks (HAE A+) and those without (HAE A−). *p < 0.05 HAE A+ vs. healthy controls (CTR). § HAE A− vs. CTR.

Figure 3 shows the details of the comparison between the HAE A+ vs. HAE A−, HAE A+ vs. CTR, and HAE A− vs. CTR groups in terms of emotion dynamics.

The main finding is that the HAE A− group has higher μ_PA_ (HAE A− 1.2 [0.3] vs. CTR 0.1 [1.0], *p* = 0.021) and μ_VA_ (HAE A− 1.9 [0.8] vs. CTR 0.3 [0.7], *p* = 0.002) than the CTR group. This difference is also confirmed when looking at individual item scores, as shown in Table 3, where all PA (*p* < 0.03) and VA (*p* < 0.01) items are significantly higher in the HAE A− group, as are two NA items (μ_NA2,_ *p* = 0.01; μ_NA4,_ p = 0.02).

**Table 3:** Between groups comparison of single items mean scores.

Items		Label	HAE A+	HAE A−	HAE-C1INH	CTR
Positive affect	μ_PA1_	No Energy/Full of Energy	−0.04 [0.9]	1.3 [0.4]^c^	0.5 [1.4]	0.2 [1.3]
	μ_PA2_	Tired/wide awake	−0.2 [0.7]^b^	1 [0.5]^c^	0.4 [1.3]	0.1 [1.4]
	μ_PA3_	Listless/highly motivated	0.2 [1]	1.3 [0.7]^c^	0.6 [1.2]	0.3 [0.9]
	μ_PA4_	Bored/enthusiastic	0.1 [0.6]	0.8 [0.7]^c^	0.5 [1.1]	0.4 [0.5]
Negative affect	μ_NA1_	Relaxed/stressed	0.7 [1.8]	−0.5 [0.6]	−0.5 [1.8]	0.4 [0.8]
	μ_NA2_	Peaceful/angry	−0.4 [1.7]	−1.4 [0.4]^c^	−1 [1.2]	−0.4 [0.6]
	μ_NA3_	Calm/nervous	−0.2 [1]	−1.4 [0.7]	−0.8 [1.5]	0.04 [0.7]
	μ_NA4_	Free of worry/worried	0.3 [1.8]	−0.3 [0.6]^c^	−0.2 [1.4]	0.9 [1.4]^a^
Valence	μ_VA1_	Dissatisfied/satisfied	0.9 [1.3]	2 [0.6]^c^	1.3 [1.3]	0.2 [0.8]^a^
	μ_VA2_	Unhappy/happy	0.7 [1.3]	1.7 [0.8]^c^	1 [1.2]	0.3 [0.7]^a^

Data are reported as median [IQR]. HAE A+, Patients with hereditary angioedema with attacks; HAE A−, Patients with hereditary angioedema without attacks; HAE-C1INH, Patients with hereditary angioedema due to C1INH deficiency; CTR, healthy controls. μ, mean score. ^a^*p* < 0.05 HAE-C1INH vs. CTR; ^b^HAE A+ vs. HAE A−; ^c^HAE A− vs. CTR.

There are no differences between HAE A+ and CTR. However, there is a significant difference between HAE A+ and HAE A− on the μ_PA2_ item (*p* = 0.042).

The $\sigma^{2}$ and MSSD of each specific item do not show significant differences between the three groups.

### Correlations between affect and AE-QoL questionnaire in HAE-C1INH

Correlation analyses between emotion dynamics and AE-QoL for HAE-C1INH patients are shown in Table 4. The μ_PA_ shows a strong negative correlation with the total AE-QoL score ( ρ = − 0.78, *p* = 0.003), and almost all of its subscales: fatigue/mood ( ρ ≤ − 0.69, *p* ≤ 0.013), fears/shame ( ρ ≤ − 0.58, *p* ≤ 0.049) and food ( ρ ≤ − 0.62, *p* ≤ 0.033). The μ_VA_ is also negatively correlated with the total AE-QoL score ( ρ = − 0.68, *p* = 0.014), which is mainly driven by the fatigue/mood domain of the AE-QoL (μ_VA_; ρ = − 0.65, *p* = 0.022). No significant correlations were found between NA features and AE-QoL.

**Table 4:** Correlations between EMA measures and AE-QoL in patients with HAE-C1INH.

EMA	Measures	AE-QoL		AE-QoL functioning		AE-QoL fatigue/mood		AE-QoL Fears/Shame		AE-QoL Food	
		*ρ*	*p*-value	*ρ*	*p*-value	*ρ*	*p*-value	*ρ*	*p*-value	*ρ*	*p*-value
Positive activation	μ_PA_	**−0.78** ^**a**^	**0.003**	−0.46	0.133	**−0.69** ^**a**^	**0.013**	**−0.58** ^**a**^	**0.049**	**−0.62** ^**a**^	**0.033**
	σ^2^_PA_	−0.18	0.586	0.06	0.843	0.07	0.826	−0.23	0.466	**−0.62** ^**a**^	**0.033**
	MSSD_PA_	−0.33	0.296	−0.06	0.865	−0.11	0.724	−0.33	0.303	−0.57	0.053
Negative activation	μ_NA_	0.43	0.158	0.37	0.240	0.26	0.407	0.35	0.266	0.24	0.455
	σ^2^_NA_	−0.40	0.194	−0.06	0.865	−0.28	0.375	−0.37	0.241	−0.51	0.087
	MSSD_NA_	−0.43	0.158	−0.06	0.865	−0.28	0.375	−0.42	0.178	−0.56	0.058
Valence	μ_VA_	**−0.68** ^**a**^	**0.014**	−0.40	0.204	**−0.65** ^**a**^	**0.022**	−0.54	0.070	−0.43	0.161
	σ^2^_VA_	0.10	0.778	0.40	0.193	−0.11	0.732	0.06	0.862	0.12	0.712
	MSSD_VA_	−0.12	0.704	0.15	0.649	−0.11	0.724	−0.15	0.654	−0.14	0.670

*ρ*, Spearman’s Rho correlation coefficient; *p*, *p*_value; HAE-C1INH, Patients affected by hereditary angioedema due to C1INH deficiency; μ, mean; σ2, Variance; MSSD, Mean of squared successive differences; PA, Positive Activation; NA, Negative Activation. VA, Valence; AE-QoL, AngioEdema Quality of Life. ^a^*p* < 0.05.

## Discussion

This study is the first to explore the affective dynamics of patients with HAE-C1INH, a rare genetic disease that significantly affects QoL, using the EMA approach and comparing their emotional patterns to those of healthy controls. Our findings provide novel insights into the emotional experiences of individuals with HAE-C1INH and highlight the potential of EMA as a complementary tool to traditional QoL assessments.

The HAE-C1INH and CTR groups were comparable in terms of sociodemographic variables and showed no significant difference in anxiety and depressive symptoms, even if we noticed that the PHQ-2 median score in the CTR group reaches the cut-off point for the presence of mild depressive symptoms (Giuliani et al., 2021). The lack of significant differences between the two groups suggests that, despite the presence of a rare and chronic disease, patients with a good disease control are not more susceptible to psychological symptoms than CTR.

### Emotional patterns in HAE-C1INH and CTR

Regarding the primary aim of the study, patients reported higher levels of valence (μ_VA_) (i.e., greater perceived emotional pleasantness) and lower levels of concern (μ_NA4_) compared to CTR. Such patterns suggest that individuals with HAE-C1INH might have developed adaptive emotional responses, potentially as a coping mechanism. Chronic diseases often lead to psychological distress; however, resilience and psychological adaptation can mitigate their emotional burden. The higher valence scores in patients may reflect an adjustment process, possibly related to the effective disease management (Zarnowski et al., 2021; Christiansen et al., 2015) resulted from the high AECT scores, wherein individuals reframe their experiences and develop positive coping strategies to maintain emotional well-being. These findings are in line with previous research using EMAs and/or other instruments for affect measures in different chronic conditions, such as rheumatoid arthritis and asthma (Stone et al., 1997; Smyth et al., 2014) in which a relatively stable positive affect coexists with sudden peaks of negative responses triggered by disease unpredictability (Zautra et al., 2001). Moreover, according to the Response Shift Theory (Howard et al., 2011; Sprangers and Schwartz, 1999), individuals with chronic illnesses often adapt to their condition through various mechanisms including: redefining standards (recalibration); redefining meaning (reconceptualization); reprioritizing values (reprioritization). This counter intuitive pattern may be partially accounted for by the between-group difference in depressive symptoms. The CTR group scored at the threshold for mild depressive symptoms on the PHQ-2. Since anhedonia (a reduced capacity to experience positive emotions) is a core feature of depression, is strongly associated with reduced positive affect (Bylsma et al., 2008) and is one of the two domains directly measured by the PHQ-2, the lower positive affect and valence observed in controls may reflect their subclinical depressive symptoms. Regarding affect variability and instability, both groups exhibited relatively stable affective patterns, with no significant differences. It is known that variations in affect are closely linked to QoL, anxiety and depression, overall distress, and physical health (Varma, 2017; Watson et al., 1988; Steptoe et al., 2005; Naragon-Gainey, 2019; Barrett and Bliss-Moreau, 2009), however our findings suggest that these fluctuations may not be a defining feature of HAE-C1INH. Affective states may also act as precursors to these psychological conditions, and can be potentially used for evaluating the influence of both the onset and progression of disease-related symptoms (Barrett and Bliss-Moreau, 2009). The absence of between-group differences indicates that having a chronic and rare disease does not necessarily lead to increased affective fluctuations, suggesting the involvement of the already mentioned modulatory factors.

### The role of HAE-C1INH attacks in affect regulation

As for the secondary aims, HAE A− group showed a significantly higher score in the composite measure of Positive Activation (μ_PA_) and Valence (μ_VA_), while exhibiting lower levels of μ_NA2_ (peaceful/angry) and μ_NA4_ (free of worry/worried) compared to CTR. These findings indicate that individuals without attacks reported an overall more positive affective profile compared to CTR, characterized by heightened pleasantness, energy and engagement. Concomitantly, the reduced levels of μ_NA2_ and μ_NA4_ indicate a lower prevalence of negative emotions such as worry and anger. A possible explanation for these unexpected results may lie in the efficacy of the treatment, which is known to improve both psychological symptoms and QoL (Lumry et al., 2014), and may have played a positive role in the patients’ affective profile, positioning them at an even better level than CTR when the disease is fully under control.

When comparing HAE A− and HAE A+, the latter had significantly lower μ_PA2_ (tired/wide awake), indicating reduced energy levels. This difference may be partially explained by the presence of fatigue, which is a common prodrome and consequence of HAE-C1INH attacks (Jean-Baptiste et al., 2022; Prematta et al., 2009) and may have negatively affected perceived energy levels.

### EMA as a complementary tool for QoL assessment

The correlation analyses performed in the HAE-C1INH group revealed moderate to strong negative associations between the μ_PA_ and μ_VA_ with QoL scores, particularly in the fatigue/mood and fears/shame domains. These data show that high-activation positive emotional states are associated with better QoL among HAE-C1INH patients (Beard et al., 2022). Because the analysis is a correlation, the direction of this relationship cannot be established. Positive affect may contribute to a better QoL, and a good QoL may foster positive affect or the two may reinforce each other.

Moreover, the present findings also suggest that traditional QoL measures, which primarily focus on disease burden, may overlook key emotional resources and coping mechanisms developed by patients. EMA of affect, by capturing real-time emotional states, provides a more nuanced understanding of how patients experience their condition on a daily basis.

This pattern indicates that the AE-QoL is sensitive to capturing the degree of well-being associated with enhanced/diminished positive affective states (VA and PA). However, at the same time it appears less equipped to capture variations in negative affective states (NA). The AE-QoL may not fully encompass the breadth or intensity of negative affective states but can accurately reflect fluctuations linked to positive affect. We chose the AE-QoL rather than the disease-specific HAE-QoL (Prior et al., 2012) for reasons of comparability and consistency: the AE-QoL is the instrument routinely used within the ITACA registry, to which our patients belong, and it is the most widely adopted QoL measure in recent angioedema research and clinical trials.

### Clinical and research implications

Our findings highlight the importance of considering emotional resilience and positive affect in the management of HAE-C1INH, as well as the potential of the EMA approach to gain a more comprehensive understanding of the patients’ emotional experiences. Interventions aimed at enhancing adaptive coping mechanisms such as cognitive reappraisal, acceptance and mindful-based techniques, problem-focused coping and psychoeducation, could further support patient well-being. Additionally, future research should explore whether similar affective patterns emerge in other chronic diseases and whether EMA-based interventions can be leveraged to promote psychological well-being.

### Limitations and future directions

Despite the innovative nature of this study and the use of EMAs, several limitations should be considered, and results interpreted with caution. First, the small sample size and single-centre design reduce the generalizability of the findings and may have limited statistical power, especially in subgroup analyses and group comparison, increasing the risk of Type II errors. Moreover, given the exploratory and hypothesis-generating nature of the study, no formal correction for multiple comparisons was applied; Type I errors cannot be excluded and interpretation of our findings should be cautious.

Although no statistically significant age difference emerged between groups, a disparity in median age was present and analyses did not account for confounders. This may have subtly influenced emotional patterns, particularly given the known associations between age and affect dynamics (Carstensen et al., 2011; Puente-Martínez et al., 2021). Similarly, although the sex distribution was comparable between groups and did not differ significantly, the limited sample size precluded a meaningful analysis of gender effects on affect dynamics. Given that emotion regulation varies by biological sex (Poláčková Šolcová and Lačev, 2017), this represent a further variable that future powered studies should account. Second, the observational design and limited timeframe prevent the inference of causal relationships between affective states and attacks. The contribution of the attacks as well as LTP to the affective dynamics was not accounted for. While EMA offers the advantage of minimizing recall bias and enhancing ecological validity, our weekly sampling schedule, chosen to balance data richness with participant compliance, may have overlooked finer-grained affective fluctuations occurring throughout the day or in proximity to attacks. In this study, we prioritized observation length over sampling intensity to reduce participant burden and ensure adherence over 16 weeks, therefore our design is characterized by a low-frequency sampling. Future research could benefit from more frequent or event-based EMA designs to better capture dynamic emotional changes during different illness phases. A further limitation concerns the AECT and AE-QoL, which have a 4-week recall window and, in our protocol, were administered only at baseline. They therefore captured a different and shorter time frame than the 16 weeks covered by EMAs, which limits their direct comparability with the EMA data. Additionally, although participants were given access to an online registry (ITACA) to report the frequency, duration, and severity of attacks, this data was inconsistently completed and could not be reliably analyzed. This represent a significant limitation, not only for this study but for future ones more focused on attacks. Therefore, the development of more user-friendly digital diaries or passive monitoring systems may improve symptom tracking in future studies. Lastly, it is important to acknowledge that our sample may represent a subgroup of highly engaged and adherent patients, more prone to digital participation potentially contributing to the unexpectedly positive emotional profiles observed. This potential selection bias further limits the generalizability of the findings to the broader HAE-C1INH population. Integrating physiological measures, such as heart rate variability, could provide additional insight into the biological underpinnings of emotional regulation in this population.

## Conclusion

This study underscores the potential of EMA in capturing real-time emotional states and enhancing the assessment of QoL in HAE-C1INH patients. The observed higher valence and lower worry levels challenge conventional assumptions about frailty, suggesting that adaptive mechanisms play a crucial role in shaping emotional experiences. This dynamic approach not only complements traditional QoL evaluations, but also has the potential to uncover novel intervention strategies. Understanding the temporal patterns of affective states in relation to HAE-C1INH could inform personalized coping mechanisms and enhance disease management, ultimately leading to more targeted and personalized interventions.

## Data Availability

The datasets presented in this study can be found in online repositories. The names of the repository/repositories and accession number(s) can be found at: the dataset used and analyzed during the current study are available in Figshare with the following identifier: https://doi.org/10.6084/m9.figshare.29148740.v1.
